# Different graphene layers to enhance or prevent corrosion of polycrystalline copper†

**DOI:** 10.1039/c8ra00412a

**Published:** 2018-04-23

**Authors:** Ying Xu, Jingyi Qu, Yongtao Shen, Wei Feng

**Affiliations:** School of Materials Science and Engineering, Tianjin University, Tianjin Key Laboratory of Composite and Functional Materials Tianjin 300072 P. R China weifeng@tju.edu.cn shenyt@tju.edu.cn; Collaborative Innovation Center of Chemical Science and Engineering (Tianjin), Tianjin Key Laboratory of Composite and Functional Materials Tianjin 300072 P. R China; Key Laboratory of Advanced Ceramics and Machining Technology, Ministry of Education Tianjin 300072 P. R China

## Abstract

Graphene was used as an anticorrosive coating for metals as it can effectively isolate the corrosion factors such as oxygen. However, we found that the anticorrosive and corrosive effects on metal surface were related to graphene layers and metal crystal faces. In this paper, we found that different layers of graphene had significantly different effects on the corrosion of polycrystalline copper during long-term storage under atmospheric conditions. Optical images and Raman spectra showed that single layer graphene (SLG)-coated copper had a higher degree of corrosion than bare copper. However, when covered with CVD *in situ*-grown bilayer graphene (BLG), the copper foil was effectively prevented from being etched as it exhibited a bright yellow color despite the differences in crystal faces. The surface potential differences measured by an electric force microscope (EFM) showed that a contact potential difference (*V*_CPD_) between 30 and 40 mV existed between Cu/SLG and bare copper. The SLG-coated areas had a higher surface potential (SP), which meant that the (SLG)-coated copper was more prone to lose electrons to exhibit galvanic corrosion. The BLG coating made SP of underlying copper lower making it harder to lose electrons; thus, BLG successfully protected the copper from being corroded. These findings have a foreseeable significance for graphene as a metal anti-corrosion coating.

## Abstract 2

The degree of corrosion depends on the crystal faces and number of graphene layers, whereas BLG can be used as an anticorrosion coating.

## Graphical abstract

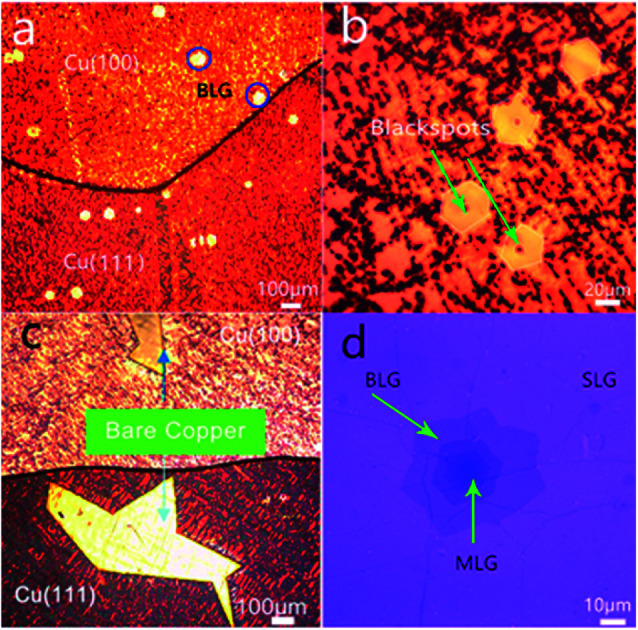

## Introduction

Graphene was once considered to be an excellent anti-corrosion agent for metals under relatively harsh conditions over a short time scale,^1–3^ and it can slow down the electrochemical corrosion rate significantly in a solution environment.^4,5^ However, when stored at room temperature for a long time in ambient environment, some researchers found that graphene could not protect the metals from corrosion and also played a role in accelerating their oxidation.^6–9^ They attributed the enhanced corrosion property of graphene to its ability to promote electrochemical corrosion of copper. However, the current proposed mechanisms have not focused on the influence of the number of graphene layers and grain orientations of polycrystalline copper on copper corrosion. Herein, we reported the protection and corrosion mechanisms of polycrystalline copper coated with CVD single layer graphene coexisting with multilayer graphene. We found that regardless of the crystal face of the copper surface, it corroded faster in the presence of the single layer graphene (SLG) coating compared with bare copper without a graphene coating. Besides, when coated with pure bilayer graphene (BLG), the polycrystalline copper effectively avoided being corroded regardless of the crystal face of copper. The mechanisms of corrosion and protection were studied by an electric force microscope (EFM), which could detect the surface potential (SP) differences and then deduced the contact potential differences (*V*_CPD_); this provided a driving force for galvanic corrosion. *V*_CPD_ between Cu/SLG and bare copper was 30–40 mV, which represented a driving force for galvanic corrosion. Meanwhile, BLG successfully protected the copper, since BLG coating made SP of underlying copper lower, which meant that it was harder to lose electrons than surrounding SLG coating regions. Our research showed that the use of BLG as an anticorrosive coating of copper under room temperature is strongly effective.

## Results and discussion

### Single-layer graphene (SLG) as a corrosion promoter of copper under long-term ambient conditions

**Fig. 1:**
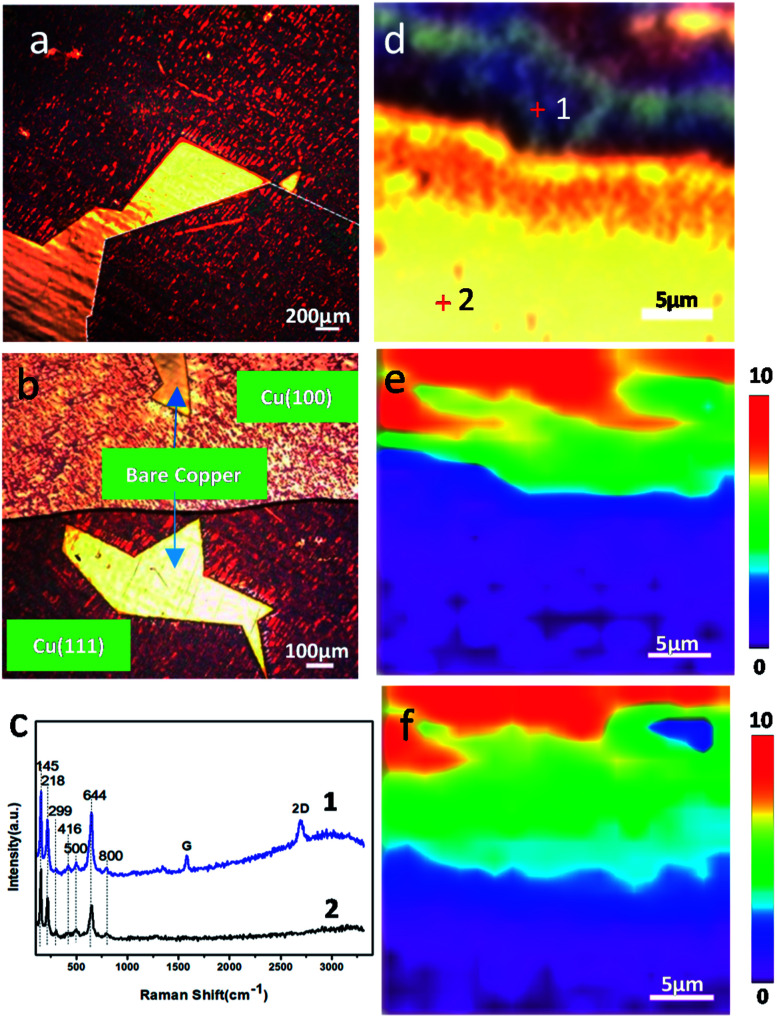
(a) Optical images of Cu/graphene and (b) SLG coated Cu (111) and (100). (c) Raman spectra obtained at positions marked in optical image (d) and (e) Raman maps of FWHM_2D_ and (f) FWHM_644_ corresponding to (d).

To study the dependences of corrosion and anticorrosion mechanisms of graphene-coated copper on the number of graphene layers and metal crystal faces over a long time scale, a copper foil with CVD graphene grown on a large area was stored at ambient environment in room temperature for up to 18 months. Fig. 1 shows the optical images of the CVD graphene-coated copper after a long-term air exposure at room temperature. The corresponding Raman spectrum (Fig. 1c) and Raman maps (Fig. 1e) indicated the presence of graphene. Fig. 1a shows that the size of the SLG domain could reach to millimeters; it could cover most of the area of the copper surface while leaving a small area of bare copper directly exposed to ambient conditions. In addition, a drastic color change from yellow to red and purple was observed as shown in Fig. 1a and b, which was attributed to the oxidations on its surface.^9–11^ We observed that SLG played a role of promoting corrosion regardless of the crystal face of the copper. The full width at half maximum of 2D bands of FWHM_2D_ map of the sample proved the presence and position of graphene on copper (Fig. 1e) and confirmed that the bright yellow area in optical images was bare copper. Besides, the width and shape of the 2D band confirmed the number of graphene layers.^12,13^ The Raman spectrum of Cu/SLG (Fig. 1c) showed multiple peaks between 100 cm^−1^ and 800 cm^−1^, corresponding to various copper oxides: Cu_2_O (145 218 416 644 cm^−1^), CuO (299, 500 cm^−1^), and Cu(OH)_2_ (800 cm^−1^),^10,14^ indicating that copper underneath SLG had been corroded during long-term and room temperature storage. Also, the Raman spectrum obtained from bare copper far away from the graphene boundary (Fig. 1c) similarly exhibited the peaks of copper oxides combined with the presence and position of SLG (Fig. 1e) and distribution of the characteristic peak of Cu_2_O (FWHM_644_) in Raman map (Fig. 1f); therefore, we could conclude that the content of copper oxides depended on the presence of graphene. This result strengthened the evidence that the presence of SLG promotes corrosion.

### Effect of the crystal faces on the corrosion rate of copper

**Fig. 2:**
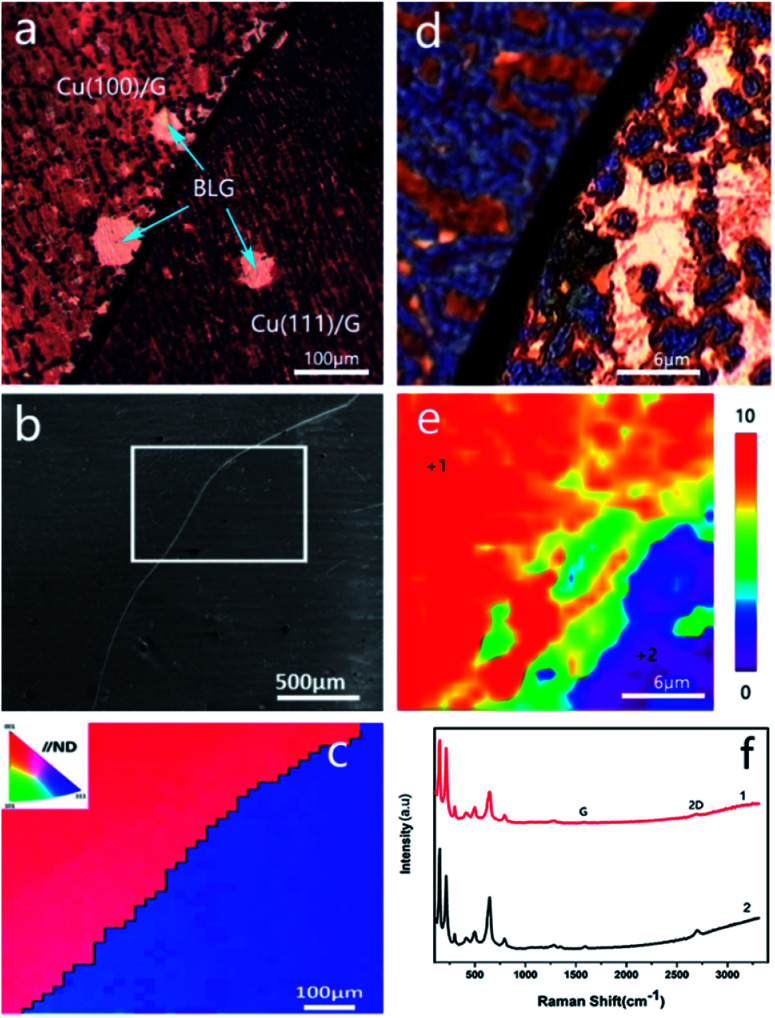
(a) Optical image of graphene-coated Cu. (b) SEM image of the corresponding area of (a). (c) Corresponding IPF map from the white frame area of (b). (d) Optical image of the Raman map area. (e) Combined FWHM_644_ and (f) Raman spectra obtained at positions marked in (e).

The effect of grain orientations on the corrosion rate of graphene-coated copper was studied by electron backscatter diffraction (EBSD), which could detect the texture of the polycrystalline copper.^15^ The EBSD map is shown in this study as an inverse pole figure (IPF) map (Fig. 2c); in addition, the color of each grain stands for the crystal orientation parallel to the sample surface normal, which is commonly noted as the sample ND direction. For this sample, the red color represented the Cu (100) plane, and the blue color was for the Cu (111) plane (Fig. 2c). Fig. 2b shows a scanning electron microscopy image (SEM) of graphene-coated copper with a gray line, which was the grain boundary between Cu (100) and Cu (111); from the long grain boundary and its IPF map we could conclude that the sample was polycrystalline. A copper domain with a clear black boundary that resulted from the intergranular corrosion was observed as shown in Fig. 2a.^16^ From the color of the optical image we could conclude that the Cu (111) plane had a deeper color than Cu (100) plane; thus, we suspected that in this bi-crystal material, the corrosion rate of 〈111〉//ND orientation was faster than that of 〈100〉//ND orientation. The Raman spectra (Fig. 2f) obtained at different crystal faces exhibited characteristic peaks of copper oxides and did not display obvious differences in the types of peaks (Fig. 2f), whereas the distribution of the characteristic peak of Cu_2_O (FWHM_644_) in Raman map (Fig. 2e) confirmed the conclusion that Cu (111) was more reactive than Cu (100).

**Fig. 3:**
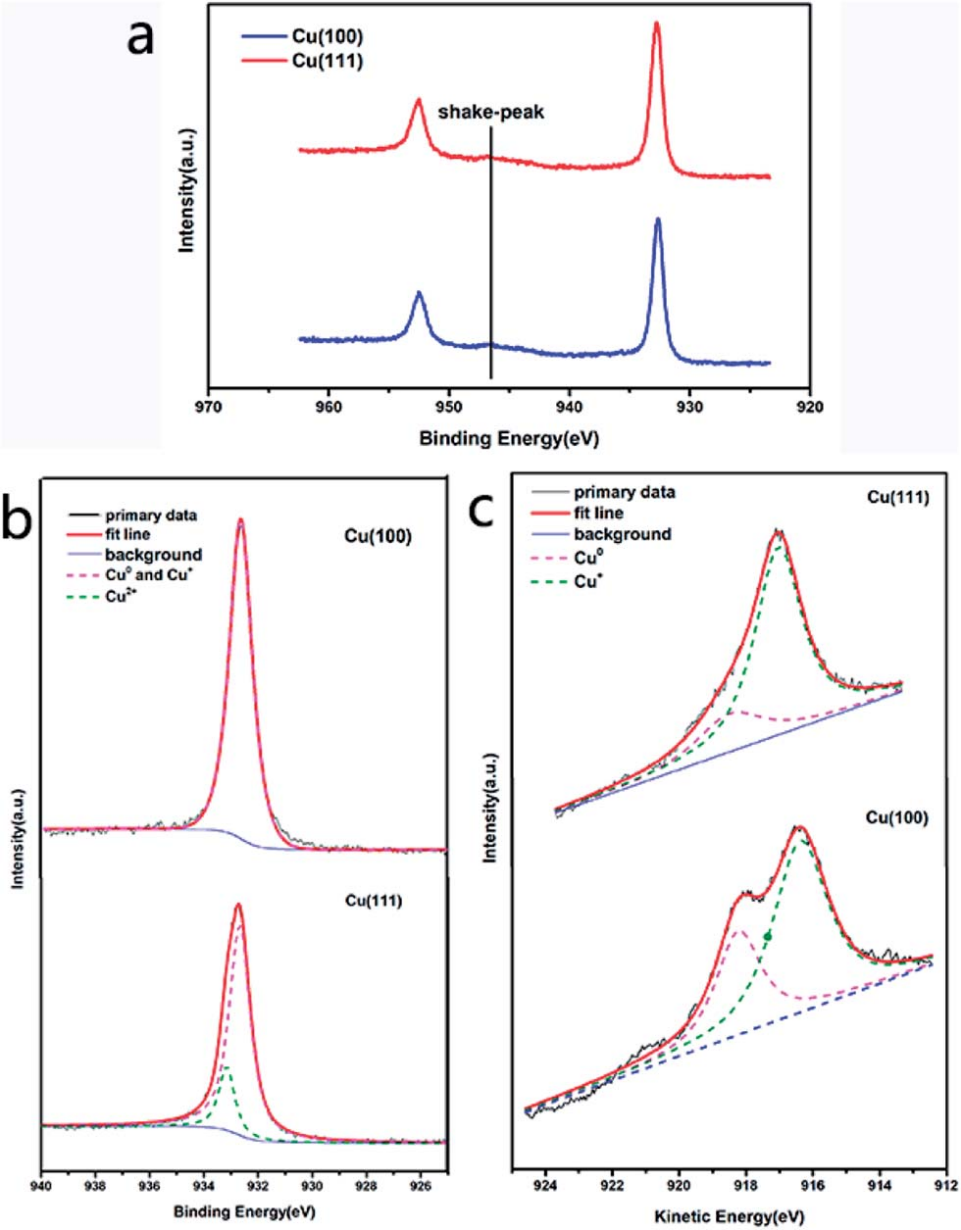
The Cu 2p spectra (a), Cu 2p_3/2_ spectra (b) and kinetic energy spectra of the Auger LMM electron (c) of Cu (100) and Cu (111).

Analysis of Cu 2p and Cu 2p_3/2_ spectra of Cu (100) and Cu (111) also confirmed our conclusion that Cu (111) plane easily corroded under long-term, room temperature storage relative to Cu (100) plane when coated with graphene. The presence of the shake-up peak and a higher Cu 2p_3/2_ binding energy (933.0–933.8 eV) are two major XPS characteristics of CuO, whereas a lower Cu 2p_3/2_ binding energy (932.2–933.1 eV) and the absence of the shake-up peak are characteristics of Cu^0^.^17^ The Cu 2p XPS spectra of both Cu (100) and Cu (111) contained a shake-up peak of weak intensity at 946 eV (Fig. 3a), and the Cu 2p_3/2_ peak was centered at about 932.7 eV. In the Cu 2p_3/2_ spectrum of Cu (100), the characteristic peak of CuO (933.0–933.8 eV) (Fig. 3b) was hardly visible, whereas the Cu 2p_3/2_ spectrum of Cu (111) (Fig. 3b) showed a characteristic peak of CuO with a binding energy of 933.2 eV, which indicated that Cu (111) had a higher content of CuO than Cu (100). However, the Cu 2p_3/2_ binding energies could not be used to distinguish Cu_2_O and CuO because they were essentially identical. The Auger LMM lines of Cu were used to solve this problem. It was observed in Fig. 3c that the broad feature in the kinetic energy spectra of the Auger LMM electron consisted of contributions from two copper species (Cu^+^ and Cu^0^). The peaks in the Auger kinetic spectra (916–916.5 eV for Cu^+^ and 917.8–918.3 eV for Cu^0^) must correspond to Cu^+^ and Cu^0^ species.^17^ Their various spectra were normalized, and the intensity ratios were determined for semi-quantitative analysis. The results of this analysis are summarized in Table 1. We could conclude that the ratio of Cu^+^/Cu^0^ of Cu (111) was higher than that of Cu (100), suggesting that Cu (111) had a higher content of Cu^+^, which was consistent with our Raman map results.

**Table 1:** Oxidation states of different crystal faces of copper/G after long-term room temperature storage as determined from LMM Auger spectra

Crystal face	The spectral area ratio of Cu^+^/Cu^0^
Cu (100)	1.9638
Cu (111)	3.4430

These phenomenon and measurements commonly indicate that the corrosion rate of graphene-coated copper corresponds to the grain orientations of copper, and Cu (111) is more reactive than Cu (100).

While this outcome is contrary to the results of the surface energies because they have conspicuous differences in different crystal faces with the close-packed plane (111) having the lowest surface energy, which means it has the lowest chemical activity.^18^ Thus, the ranking of low-index crystal planes of copper for chemical reactivity is (111) < (100), and it also positively correlates with the reactivity of corrosion.^19^ Consequently, in terms of surface energies, the (111) plane should be more resistant to corrosion than the (100) plane, which is inconsistent with our experimental results. We assume that in addition to the specific grain orientations and surface energies, the misorientation between adjacent grains is also a critical fact in this phenomenon. The divergence in corrosion behaviors of the distinct oriented grains is likely to result from the anodic/cathodic nature of the crystal faces.^20,21^

### Bi-layer graphene (BLG) as an anti-corrosion coating of copper in long-term, room temperature storage

**Fig. 4:**
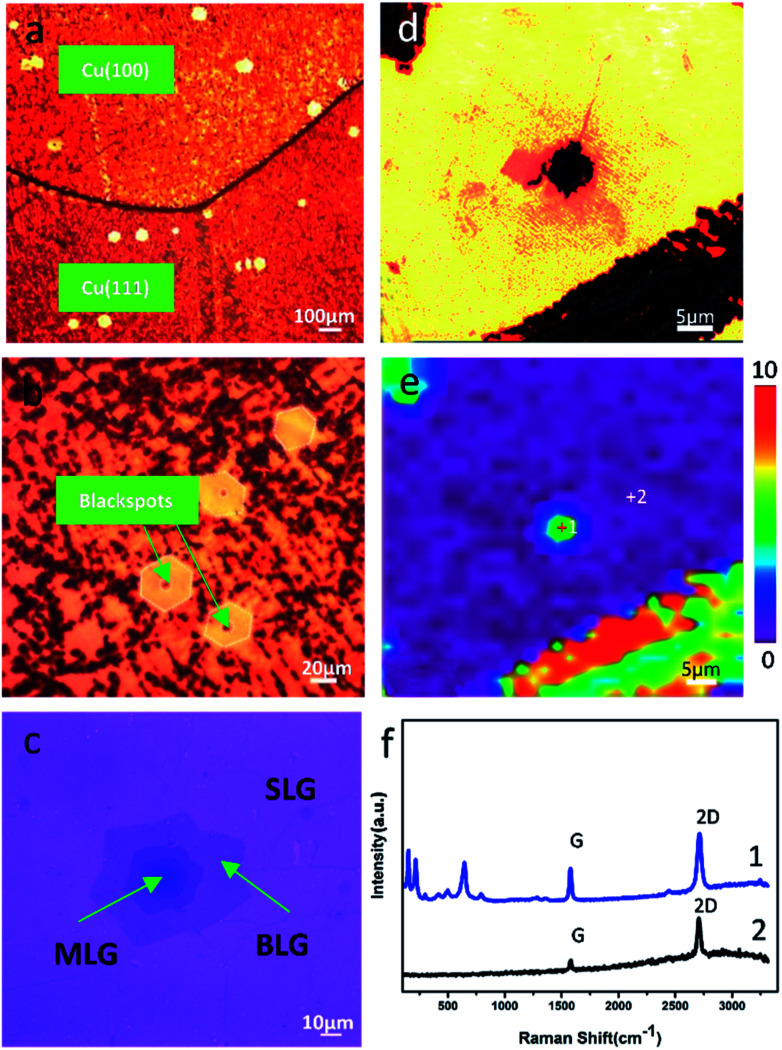
(a) Optical image of the graphene-coated copper with some BLG-coated regions. (b) The magnified optical image with the BLG region and some central areas of BLG have blackspots. (c) Transferred graphene. (d) Optical image of the blackspot area at the center of MLG-coated copper and (e) corresponding Raman map of FWHM_644_ and (f) Raman spectra obtained at positions marked in part (e).

We found that the copper surface underneath BLG was effectively protected regardless of whether it was the (111) plane or the (100) plane. The existence of some bright small hexagons on the polycrystalline copper was observed from the optical images (Fig. 4a and b). We put forward that BLG could validly protect copper from corrosion in long-term, room temperature storage. This assumption was proved by the optical images (Fig. 2a and 4a, Fig. S4†) in which the bi-layer graphene-coated areas displayed a bright yellow color, indicating a little copper oxide surface; the Raman spectrum obtained from the BLG area (Fig. 4f) only displayed some characteristic peaks of graphene instead of any peaks of copper oxide. The corresponding Raman map of FWHM_644_ (Fig. 4e) strongly proved that BLG prevented the growth of copper oxide on copper under long-term, room temperature storage. Consequently, we came to a conclusion that BLG could effectively protect polycrystalline copper from room temperature corrosion.

Simultaneously, at the center of some bright little hexagon areas, a blackspot emerged. To figure out this phenomenon, we obtained Raman spectra from these blackspots as well as the centers of the bright little hexagons without blackspots (Fig. 4f and S2b†), and we found that these blackspot regions were covered with three or more layers of graphene (Fig. 4c); the Raman spectra showed the characteristic ratio of G/2D of MLG (Fig. S2b†). The optical image of the graphene that was transferred from the long-term, room temperature stored copper substrate to 300 nm SiO_2_/Si wafer clearly presents the number of graphene layers (Fig. 4c). By comparing the optical images of graphene on corroded copper with those on SiO_2_/Si wafer (Fig. S3†), we found that the corrosion of MLG-coated area was inhomogeneous since it always corroded from the center of the MLG area. Besides, the existence of a circular region of about 4 μm at the center of the Raman map of FWHM_644_ proved that the corrosion occurred in the central area. In light of the synthesis mechanism of MLG,^22^ during the CVD growth process of graphene, the high temperature annealing of Cu foil produced some defects. These defects acted as the nucleation sites for single-crystal graphene, which is proven to be copper oxide and its area becomes larger with long-term storage.^23,24^ Thus, as we observed, the copper was further corroded at the center of the MLG-covered area under long-term, room temperature storage. Meanwhile, the copper coated with pure BLG was well protected.

### Study on the mechanisms of promoting and preventing copper corrosion by SLG and BLG, respectively

On the basis of previous studies,^6–8,25,26^ we supposed that the abovementioned phenomenon may result from galvanic corrosion, since graphene has high electric conductivity and therefore can act as the cathode or anode in galvanic corrosion. Oxygen and water from the ambient environment can intrude into the Cu/SLG interface and then serve as the electrolyte to transmit electrons;^27^ therefore, graphene, oxygen, water and copper are combined into a closed loop (Fig. 6b). The optical image of the transferred graphene shows that there are some wrinkles running through the graphene (Fig. 4c), and these wrinkles are inevitable since they are formed during the cooling process.^22^ Wrinkles and other defects such as grain boundaries may provide the passageway for water and oxygen from air and would induce corrosion.^28^ As for the galvanic corrosion of SLG-coated copper, the reaction products are not generated by the direct collision reaction of oxygen molecules with copper atoms; we suggest the probable mechanisms as follows: on the anode electrode, Cu^0^ produces free electrons and transforms to Cu^2+^:

1 Cu^0^ − 2e → Cu^2+^

**Fig. 5:**
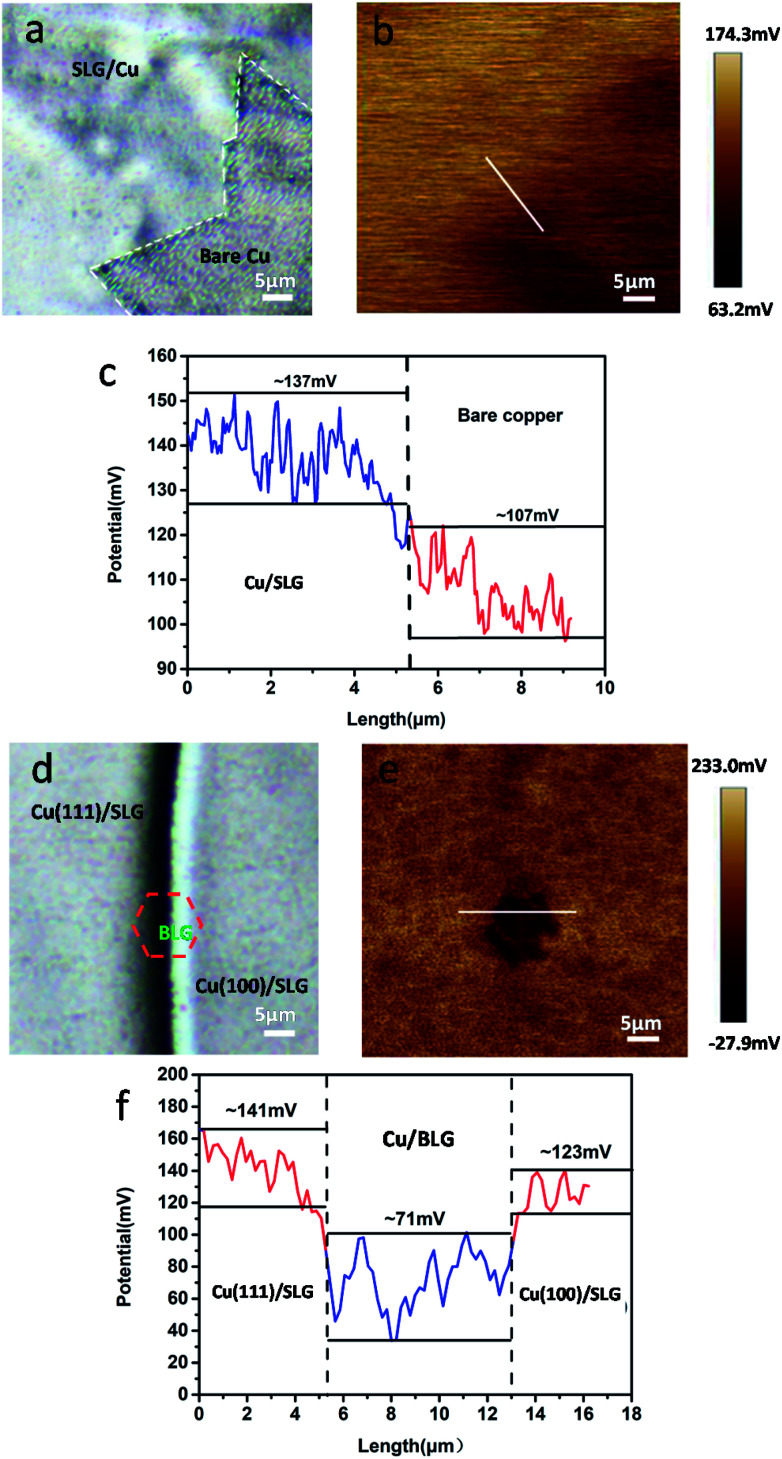
(a) and (d) Optical images of freshly prepared CVD graphene-coated polycrystalline copper. (b) and (e) are corresponding SP maps. (c) and (f) Potential profiles of b and e.

**Fig. 6:**
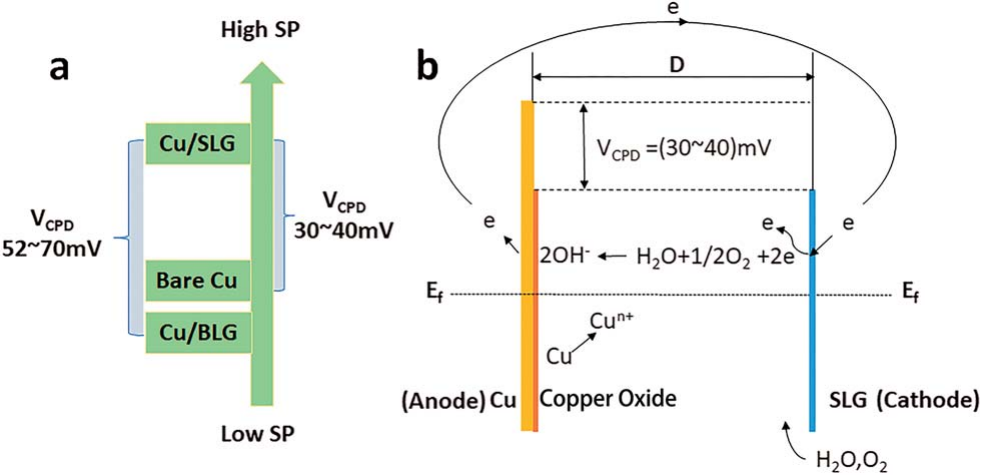
(a) The results of V_PD_ map. (b) Illustration for the mechanism of the galvanic corrosion of SLG-coated copper. *E*_f_, Fermi level; *D*, separation distance between graphene and copper. The band bending depends on the separation distance *D* from the contact.

On the other electrode (cathode), H_2_O and O_2_ adsorbed on the edges of graphene defects result from wrinkles, grain boundaries and point defects,^29^ acquire the electrons and get reduced:

2 H_2_O + 1/2O_2_ + 2e → 2OH^−^

The abovementioned reactions occur at the same time but independently and then, OH^−^ from the edge of graphene combines with Cu^2+^, generating Cu(OH)_2_ and CuO:

3 Cu^2+^ + 2OH^−^ → Cu(OH)_2_ → CuO + H_2_O

This copper oxide increases the distance between graphene and copper and thus provides more space for the transport channel of OH^−^ and transverse growth of oxide crystal nucleation. Thousands of micro-galvanic reactions undoubtedly accelerate the transfer rate of electrons. Consequently, SLG promotes the copper corrosion may attribute to its existence accelerates the transfer rate of electrons and the system satisfies all the conditions of galvanic corrosion.

To authenticate that SLG promotes the copper corrosion that stems from galvanic corrosion, whereas BLG protects copper from corrosion, we characterized SP of a freshly prepared CVD graphene sample with relatively clean copper surface *in situ* using electric force microscopy (EFM),^30^ which could detect SP. The tip was biased to zero the contact potential differences; therefore, a positive SP measured represented larger electronic activity of the sample. Potentials measured with higher and lower values with respect to the EFM tip indicated a net anodic and net cathodic activity, respectively.^31^ The potential variations of different areas applying the freshly prepared sample to exclude the influence of copper oxide were detected by EFM (Fig. 5b and e). The optical images were obtained before the detection but from the same CCD camera of EFM. To obtain SP between SLG and underlying copper, we chose the area where SLG was coated on the top left corner and bare copper was on the lower right corner. The SP images in this study were not transformed and are shown as measured. It should be recalled that lower SP corresponds to a nobler microelectrode, whereas higher SP corresponds to a less noble one. The potential values of SLG-coated areas were more positive than those of bare copper, which indicated that SLG-coated copper had higher electronic activity. Mean SP values of 137 ± 8 mV and 107 ± 6 mV were measured for SLG-coated copper and bare copper, respectively (Fig. 5c). A similar potential difference (*V*_CPD_) of 30–40 mV between Cu/SLG and bare copper indicated a driving force for galvanic interaction; hence, SLG could accelerate galvanic corrosion, whereas copper was anodic and could be corroded. The SP map with BLG-coated copper is presented in Fig. 5e. A dark little hexagon pattern displayed on the SP map represents a BLG-coated region (Fig. 5b). The SP values of BLG-coated copper (Cu/BLG) were lower than those of SLG-coated copper (Cu/SLG), and the darker color corresponded to a lower potential. The mean SP values were measured to be 71 ± 8 mV for Cu/BLG, 141 ± 5 mV for Cu (111)/SLG and 123 ± 7 mV for Cu (100)/SLG (Fig. 5f). A positive *V*_CPD_ value between Cu/SLG and Cu/BLG was 52–70 mV, indicating that Cu/SLG had a better activity than Cu/BLG, which meant that Cu/BLG remained inert in comparison to the surrounding region. This result was consistent with our experimental observation that Cu/SLG had been corroded severely, whereas Cu/BLG exhibited an almost intrinsic surface state. Accordingly, the copper underneath BLG had been protected effectively.

Besides, the mean SP of Cu (111)/SLG is higher than that of Cu (100)/SLG, and the difference in SP values provides a driving force for galvanic corrosion (Fig. 5f). This result prompts the fact that the corrosion rate of 〈111〉//ND orientation is faster than that of 〈001〉//ND orientation of copper. As for polycrystalline copper in our sample, the increased density of crystal defects leads to a decreased in WF of Cu (*W*_Cu_); thus, it is inaccurate to compare its WF with those of single crystal copper mentioned in other refs,^32,33^ and this result also increases the feasibility of the copper surface to lose an electron and be anodic.^26,34^ WF can be related to *V*_CPD_ according to the following equation:^34^

4 *V*_CPD_ = *V*_Cu/G_ − *V*_Cu_ = 1/*e*(*W*_Cu_ − *W*_Cu/G_)

Here, *W*_Cu/G_ is the work function of graphene-coated copper, *W*_Cu_ is the work function of bare copper, and *e* is the absolute value of electron charge. We get SP of Cu/G and bare copper by EFM and then deduce *V*_CPD_ as described previously. From the eqn (4), we know that higher SP means lower WF. By comparing the values of their SP (Fig. 6a), Cu/SLG has a relatively lower WF; consequently, lower energy is required to remove an electron from the Fermi level to the position just outside the sample surface in SLG-coated copper compared to that in bare copper and hence SLG-coated copper is more reactive than bare copper (*W*_Cu/SLG_ < *W*_Cu_). Likewise, WF of Cu/BLG is higher since its SP is lower than that of Cu/SLG (Fig. 6a), which means it is harder to lose electrons and be anodic for BLG-coated areas and thus, the copper underneath BLG is effectively protected.

## Conclusions

Our studies concentrate on the dependence of the protective and corrosive effects of graphene on polycrystalline copper and on the number of graphene layers. Different numbers of graphene layers have different effects on the long-term corrosion of polycrystalline copper in ambient atmosphere at room temperature. SLG promotes the corrosion of copper under a driving force of galvanic corrosion, resulting from the differences of SP, whereas BLG can protect copper from corrosion effectively regardless of the crystal face of copper because Cu/BLG has a lower SP. Simultaneously, when covered with MLG, the copper oxide nucleation sites formed during the growth of MLG grew up in long-term storage.

Besides, we observed that there were significant differences in the degree of corrosion response on 〈100〉//ND- and 〈111〉//ND-oriented grains of the graphene-coated copper, since 〈100〉//ND oriented grain was less reactive when located beside 〈111〉//ND oriented grains. Our research presented here has foreseeable implications for graphene as a metal anti-corrosion coating.

## Conflicts of interest

There are no conflicts to declare.

## Supplementary Material

RA-008-C8RA00412A-s001
