# Supplementary material for: Different graphene layers to enhance or prevent corrosion of polycrystalline copper
Source: RSC Adv. 2018 Apr 23;8(27):15181–7. doi: 10.1039/c8ra00412a (PMC9079975; doi:10.1039/c8ra00412a)
Supplement: RA-008-C8RA00412A-s001 [file RA-008-C8RA00412A-s001.pdf]

Electronic Supplementary Material (ESI) for RSC Advances.
This journal is © The Royal Society of Chemistry 2018

Supplementary information

Different Graphene Layers to Enhance or Prevent Corrosion of Polycrystalline Copper

Ying Xu[a], Jingyi Qu[a], Yongtao Shen[a]*, Wei Feng[a,b,c]*

[a]School of Materials Science and Engineering, Tianjin University, Tianjin Key Laboratory of Composite and Functional Materials, Tianjin 300072, P.R China.

[b]Collaborative Innovation Center of Chemical Science and Engineering (Tianjin), Tianjin Key Laboratory of Composite and Functional Materials, Tianjin 300072, P. R China.

[c]Key Laboratory of Advanced Ceramics and Machining Technology, Ministry of Education, Tianjin 300072, P. R China.

Experimental Section

The synthesis of graphene was carried out in a slide tube furnace using CVD. The Cu foils (127 μm thick,99.9 % polycrystalline) is commercially available. Cu foil was annealing at 1075 °C with 1000 sccm Ar and500 sccm H_2 for 2 h and was polished numerously using the chemical–mechanical polishing (CMP) method. Next, the Cu foil was placed in a quartz tube in the furnace and heated to 1045 °C with flowing 300 sccm Ar, and then the temperature was held for 4 h with flowing 300 sccm Ar and 50 sccm H_2. Graphene growth was carried out by starting the CH_4 flow at 0.5 and H_2 25 sccm with 0.5 h in the same tube furnace following completion of the annealing/reduction step of the Cu foil. The sample was cooled down quickly to room-temperature by sliding the furnace away under 300 sccm Ar and 4 sccm H_2 after growth.

Characterization

Optical microscopy images of the copper surface was conducted using an Olympus BH2-UMA in reflectance mode (Figures1a,1b,2a,3a,3b) and metallographic micros-copy images of graphene on Si/SiO2 was conducted using 6XB-011169 in reflectance mode (Figures 3c,1a,2a,S3a,S3b).

Raman maps were carried out on WITec alpha 300 RS+ confocal Raman microscopy (WITec GmbH) with 488 nm excitation laser wavelength and 600 grooves/mm grating. A diffraction-limit spot size of the 488 nm laser is obtained about 300 nm with a 100x objective (NA = 0.90). The Raman data analysis are performed with WITec Project plus software.

Corresponding Authors.

*E-mail: weifeng@tju.edu.cn (W. Feng)
*E-mail: shenyt@tju.edu.cn (Y.T. Shen)

The EBSD system was attached to an FEI field emission scanning microscope (FE-SEM) operated at 20 kV. Measurements were carried out with a step size of 20μm and the orientation data was post-processed with the commercial orientation imaging software package OIM- TSL®.

EFM provides accurate mapping of the surface potential distribution (DIMENSION icon with San Asyst, Bruker, USA). The measurements were conducted at 26℃ with 50% relative humidity with SCM-PIT of PtIr coating probe, the lift scan height is 100nm to eliminate the error caused by sample surface roughness. EFM data analysis was performed with the software NanoScope Analysis 1.5.

X-ray diffractometer (Bruke D8 Advanced 010301) employing Cu K radiation (λ =0.15418 nm). Test condition: 10-80 ^{o}, 5°/min.

X-ray photoelectron spectroscopy (ESCALAB 250Xi) reference spectra were obtained from Cu/Graphene foil after long-term, room temperature storage.

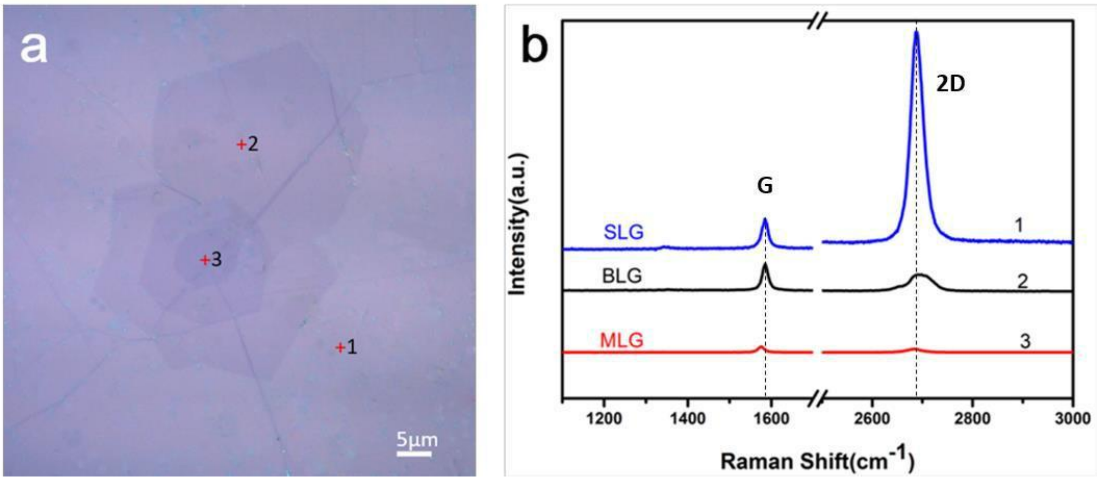

Fig. S1 (a) Optical image of pristine graphene transferred on 300 nm SiO2/Si wafer. (b) Raman spectra of graphene at positions marked in part a.

There are some wrinkles on the transferred pristine CVD graphene(Figure S1a), these wrinkles may formed during annealing process, and can provide passageway for water and oxygen from the ambient environment to intrude into the interface of graphene and copper substrates. The corresponding Raman spectra shows the high quality of pristine graphene.

Corresponding Authors.

*E-mail: weifeng@tju.edu.cn (W. Feng)
*E-mail: shenyt@tju.edu.cn (Y.T. Shen)

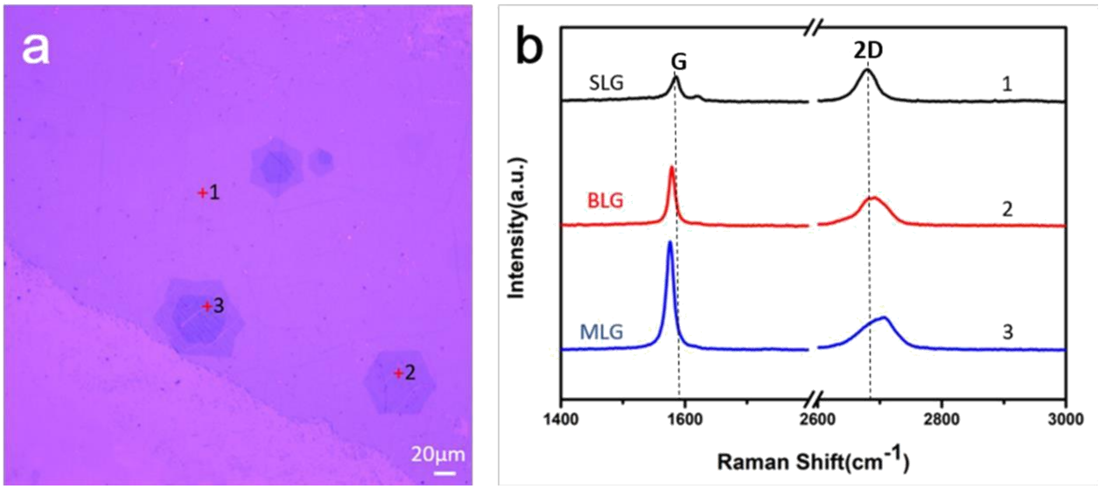

Fig.S2 Characterization of long-term, room temperature stored CVD graphene on copper and then transferred on 300 nm SiO_2/Si wafer: optical image (a), and Raman spectra (b).

The optical image and Raman spectra showed the large scale and the characteristic peaks of different numbers of CVD graphene layer, respectively. The G peak decreases strongly in intensity and shifts toward higher wave numbers with decreasing layer number. The width of the 2D line is highly sensitive to the crossover from SLG to MLG, which testified the number of graphene layers.

Corresponding Authors.

*E-mail: weifeng@tju.edu.cn (W. Feng)
*E-mail: shenyt@tju.edu.cn (Y.T. Shen)

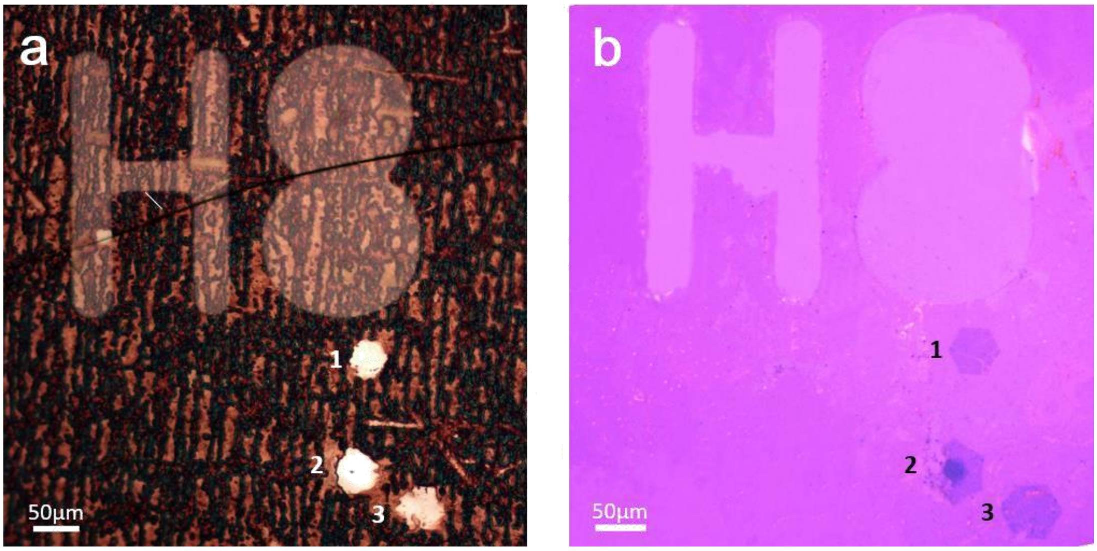

Fig. S3 Marked optical images with BLG and MLG area: (a) on corrosive copper; (b) on 300 nm SiO_2/Si wafer.

The corrosive copper were coated with gold under a templet to mark the positions of BLG and MLG, and then transferred on 300 nm SiO2/Si wafer. Position 1 was covered with a pure BLG (figure S3b), and its corresponding position showed a bright yellow color, which means the copper under BLG were protected effectively. However, at position 3, which also covered with BLG has been corroded slightly for this BLG is not integral, there are some defects may result from the CVD graphene growing process. Position 2 is a MLG coated area, the damage of it was caused by transfer process, but we can still clear recognize it is a multilayer graphene and in the center of it, a blackspot emerged, which may originated from the defects of copper during MLG growing process.

Corresponding Authors.

*E-mail: weifeng@tju.edu.cn (W. Feng)

*E-mail: shenyt@tju.edu.cn (Y.T. Shen)

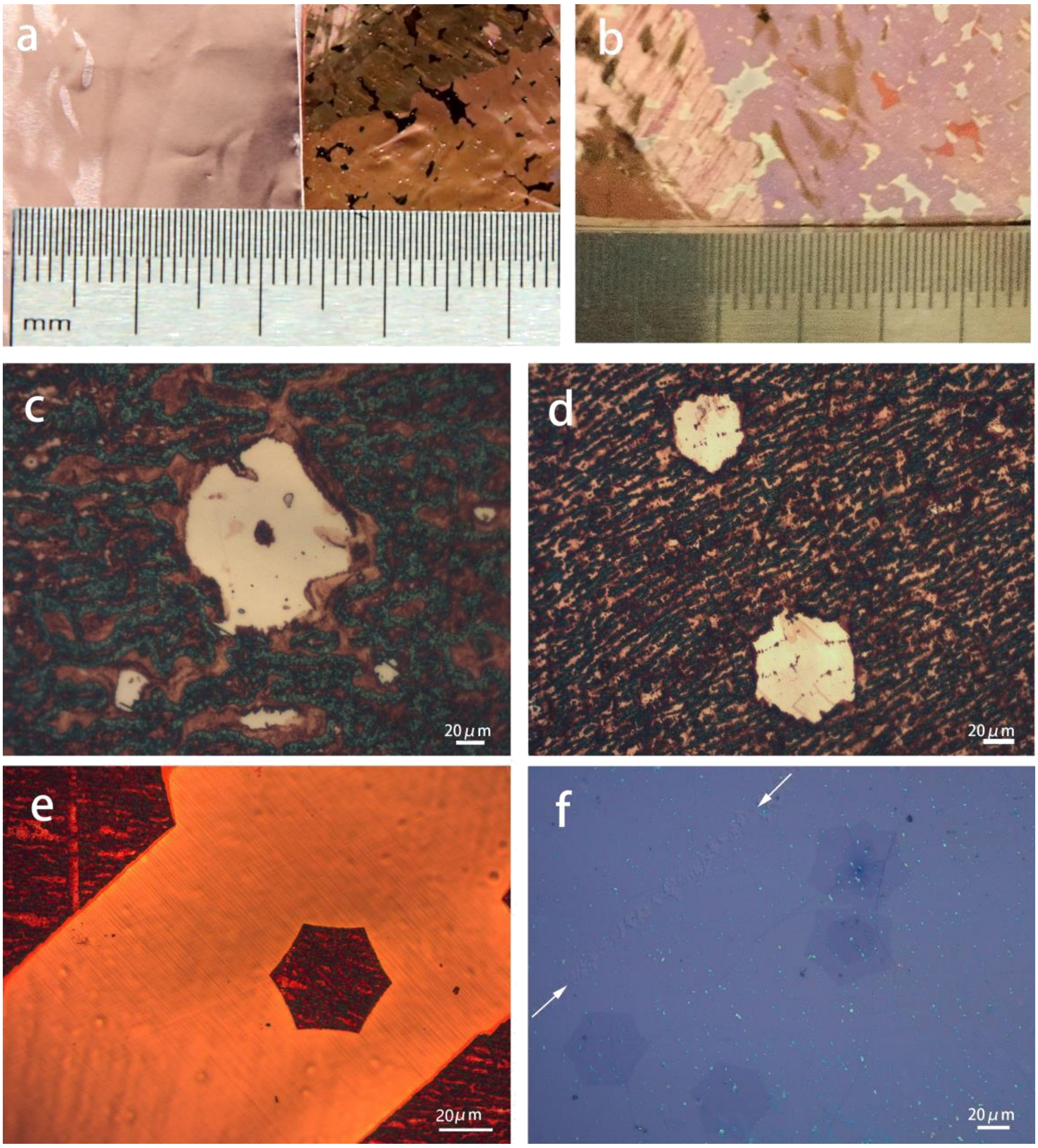

Fig. S4 Digital photos of fresh prepared copper/G and copper/G stored under long-term, room temperature condition (a) and its close-up photo

(b). Optical images of MLG coated copper (c), BLG coated area (d), Single crystal SLG grown on bare copper (e) and graphene transferred from corroded copper to SiO_2/Si wafer (f).

Comparison between fresh prepared copper/G and copper/G stored under long-term, room temperature condition, we can clearly see that after storage for almost two years, the color of copper become darker and inhomogeneous

Corresponding Authors.

*E-mail: weifeng@tju.edu.cn (W. Feng)
*E-mail: shenyt@tju.edu.cn (Y.T. Shen)

obviously suggests that it can be oxidized even with graphene coating. From fig.S4b, the close-up photo of the same sample in figure S4a, the centimeter level SLG (hexagonal structure) are visible to the naked eye because it etched the underlying copper and made it easy to distinguish from surrounding. Besides, there are two different color blocks which correspond with two different crystal faces and their corrosion differences have been distinguished by XRD and EBSD. To better understand the effects of the number of graphene layers on the corrosion of polycrystalline copper we showed the low exposure optical images of copper/G to reveal the details of the surface of the copper foil. Though there are some wrinkles run through the BLG areas which made the copper occur line corrosion, overall, combined with Figure S4c and Figure S4d, we can definitely to say, BLG can effectively protect the copper foil from long-term, room temperature corrosion. Figure S4e shows a complete single crystal SLG area, its existence proves that SLG have played a role in promoting the corrosion of copper foil for we can apparently see that the bare copper area around it is bright yellow color which is the sign of unetched copper. After transferred the graphene from corroded copper to SiO_2/Si wafer, a long line formed due to grain boundary corrosion represents damaged graphene can be seen and the number of graphene layers can be clearly identified in Figure S4f. Compare this optical image with that of the above corroded copper, the area of the BLG corresponds to the area of the hexagonal bright spot indicate that the hexagonal bright spots are caused by the BLG coating.

Corresponding Authors.

*E-mail: weifeng@tju.edu.cn (W. Feng)

*E-mail: shenyt@tju.edu.cn (Y.T. Shen)

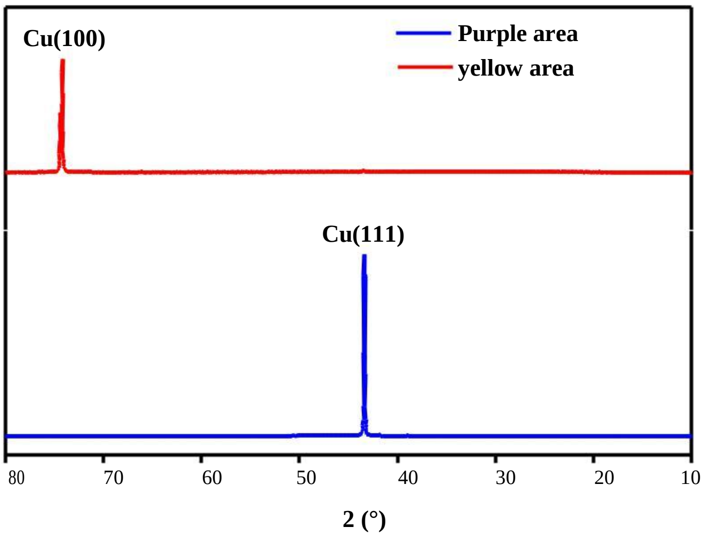

Fig. S5 X-ray diffraction patterns of graphene coated copper of Cu(100) and Cu(111)

We use XRD to confirm the crystal faces of this graphene coated polycrystalline copper sample. Fig. S5 presents the XRD spectra of the long-term, room temperature stored samples with dark purple area and yellow area. Purple area only has a Cu(111) peak indicates the main crystal face of this area is Cu(111) plane and yellow area has a Cu(100) peak which means the main component of this area is Cu(100) plane. These results consistent with IPF map tested by EBSD. However, XRD peaks due to CuO or Cu_2O were not detected in any of the samples. The absence of CuO and Cu_2O peaks may be attributed to the formation of amorphous phase under this experimental condition[1] or its oxide layer is too thin to test in XRD.

References:

1 G. Avgouropoulos and T. Ioannides, *Appl. Catal. A: Gen.*, 2003,**244**, 155–167.

Corresponding Authors.

*E-mail: weifeng@tju.edu.cn (W. Feng)
*E-mail: shenyt@tju.edu.cn (Y.T. Shen)
